# Sex Differences in Treatment Quality of Self-Managed Oral Anticoagulant Therapy: 6,900 Patient-Years of Follow-Up

**DOI:** 10.1371/journal.pone.0113627

**Published:** 2014-11-21

**Authors:** Hanna Nilsson, Erik Lerkevang Grove, Torben Bjerregaard Larsen, Peter Brønnum Nielsen, Flemming Skjøth, Marianne Maegaard, Thomas Decker Christensen

**Affiliations:** 1 Department of Cardiothoracic and Vascular Surgery & Institute of Clinical Medicine, Aarhus University Hospital, Aarhus, Denmark; 2 Department of Cardiology, Aarhus University Hospital, Aarhus, Denmark; 3 Department of Cardiology, Centre for Cardiovascular Research, Aalborg University Hospital, Aalborg, Denmark; 4 Aalborg Thrombosis Research Unit, Department of Clinical Medicine, The Faculty of Medicine, Aalborg University, Aalborg, Denmark; Maastricht University Medical Center, Netherlands

## Abstract

**Background:**

Patient-self-management (PSM) of oral anticoagulant therapy with vitamin K antagonists has demonstrated efficacy in randomized, controlled trials. However, the effectiveness and efficacy of PSM in clinical practice and whether outcomes are different for females and males has been sparsely investigated.The objective is to evaluate the sex-dependent effectiveness of PSM of oral anticoagulant therapy in everyday clinical practice.

**Methods:**

All patients performing PSM affiliated to Aarhus University Hospital and Aalborg University Hospital, Denmark in the period 1996–2012 were included in a case-series study. The effectiveness was estimated using the following parameters: stroke, systemic embolism, major bleeding, intracranial bleeding, gastrointestinal bleeding, death and time spent in the therapeutic international normalized ratio (INR) target range. Prospectively registered patient data were obtained from two databases in the two hospitals. Cross-linkage between the databases and national registries provided detailed information on the incidence of death, bleeding and thromboembolism on an individual level.

**Results:**

A total of 2068 patients were included, representing 6,900 patient-years in total. Males achieved a significantly better therapeutic INR control than females; females spent 71.1% of the time within therapeutic INR target range, whereas males spent 76.4% (p<0.0001). Importantly, death, bleeding and thromboembolism were not significantly different between females and males.

**Conclusions:**

Among patients treated with self-managed oral anticoagulant therapy, males achieve a higher effectiveness than females in terms of time spent in therapeutic INR range, but the incidence of major complications is low and similar in both sexes.

## Introduction

Oral anticoagulant therapy (OAT) with vitamin K antagonists (VKA), e.g. warfarin or phenprocoumon, remains the mainstay to prevent thromboembolism in a variety of clinical conditions. Mechanical heart valves, atrial fibrillation or recurrent venous thromboembolism are the most frequent clinical indications for long-term treatment [1]. The recent approval of new oral anticoagulant drugs (e.g. dabigatran, apixaban and rivaroxaban) for patients with atrial fibrillation has increased the number of treatment options for this patient group, however, VKA remains the cornerstone of OAT [2]. Furthermore, the investigation of the efficacy and safety of dabigatran in mechanical heart valve patients was terminated prematurely due to an excess of adverse events in the dabigatran group, leaving this patient group entirely dependent on VKA [3]. The new oral anticoagulant drugs can not be used in patients with renal impairment [4].

VKA impedes coagulation and consequently increases the risk of bleeding; hence, meticulous monitoring of coagulation time measured using the International Normalized Ratio (INR) and appropriate dosage adjustments are mandatory for patients prescribed VKA [1], [5]. General practitioners and hospital departments generally perform conventional management of VKA-therapy, but the risk of major complications continues to cause concern [6]. Patient self-management (PSM) of OAT is a concept empowering trained patients to monitor and adjust their treatment in home settings [1], [7]. Randomized, controlled trials (RCT) have demonstrated the efficacy and safety of PSM, with self-managed patients achieving a significant reduction in major tromboembolism compared to conventional monitored patients [8], [9]. The risk of thromboembolism can also be halved without a concomitant significant increase in mortality or bleeding [8]–[10]. The advantages of PSM in RCT represent the efficacy of PSM under ideal circumstances. Inclusion into a RCT is a distortion of usual practice, hence benefits shown in clinical trials might not translate into everyday clinical practice. Population-based studies evaluating clinical events are crucial for obtaining results generalizable to the general population [11], [12]. A limited number of studies have evaluated the effectiveness of PSM in clinical practice, and these follow-up studies indicate an advantage of PSM compared to conventional management [12]–[16]. However, all these studies, apart from [12] were small and/or only used surrogate endpoints.

Sex-related differences is found regarding the risk of thromboembolism and death among patients with atrial fibrillation [17]. In addition, a meta-analysis suggests that the efficacy of PSM may be sex-dependent, with males benefiting the most [10]. When compared to conventional care, males performing PSM achieve a significant reduction in thromboembolism, whereas females do not. Further investigations are important, as a considerable difference in a real-life setting may impact the approach to the educational program, which is mandatory for patients desiring to commence PSM.

Therefore, we found it interesting to investigate if differences in the quality of warfarin treatment in such patients (as reflected by TTR) exist.

The aim of this study was to evaluate the sex-dependent performance of self-managed OAT assessed by major bleeding, intracranial bleeding, gastrointestinal bleeding, stroke, systemic embolism, death and time spent within therapeutic INR target range (TTR).

## Materials and Methods

### Study design

A case-series study was conducted at two Danish centers; Center of Self-Managed Oral Anticoagulation, Department of CardioThoracic and Vascular Surgery, Aarhus University Hospital and Center of Thrombosis, Aalborg University Hospital.

The study was approved by the Danish Data Protection Agency (ref. 2012-41-0633). Ethical approval is not required for register-based studies in Denmark.

Consent from patients is not required according to Danish law and was therefore not obtained. Patient records/information was not anonymized in the databases.

### Study population

Out of approximately 3 million inhabitants in Western Denmark, an estimated 30.000 persons are prescribed VKA. General practitioners or hospital departments in Denmark referred potential eligible patients to Aarhus University Hospital in the period between 1^st^ of June 1996 and the 30^th^ of June 2012, or Aalborg University Hospital in the period between the 1^st^ of April 2008 and the 31^st^ of December 2012. All patients were required to attend an educational programme containing a minimum of three teaching lessons, including basic theoretical and practical skills including use of a coagulometer, interpretation of INR values, and VKA dosing. Over a period ranging from 3 to 27 weeks, patients gradually became self-managed. Finally, the patients were requested to demonstrate their skills in a multiple choice exam. The training scheme currently used in shown in Table 1.

**Table 1:** Self-management of oral anticoagulant therapy training program currently used.

Week number	0	3	4–15	16	> 16
Hospital INR	X	X		X	
CoaguChek INR	Daily		Weekly		
Anticipated skills	Training		Dose adjustment in cooperation between patient and the Centre	Self-management and report	
Teaching lesson	X	X		X	
Exam				X	

Abbreviations: INR: International Normalized ratio.

The only inclusion criterion for this study was patients could be regarded as capable of self-managing their OAT, and this criterion was met when the patient successfully passed the final exam. Patients regretting their decision to undertake PSM before passing the exam were not considered as self-managed and excluded from this study. Patients discontinuing PSM after passing the exam were excluded, if less than two INR measurements were reported or the time lapse between the first and second INR measurement exceeded 6 weeks.

Disabled patients and/or patients under the age of 15 could become self-managed if a caregiver or parent participated in the educational program and exam. It should be emphasized that the parent will always be involved to some extent. When we refer to the patient, therefore, this implicitly includes the parental involvement.

The TTR range was 2 – 3 for the majority of indications, such as atrial fibrillation, venous thromboembolism, thrombophilia or mechanical aortic valves and 2.5 – 3.5 for mechanical mitral valves. All patients used the portable coagulometer CoaguChek, CoaguChek S or the CoaguChek XS coagulometer (Roche Diagnostics, Switzerland) equipped with CoaguChek PT-test strips. The patients were requested to measure their INR value once a week and report all INR-data to their affiliated center.

### Outcome measures

The quality of OAT managed by PSM was assessed using both clinical and surrogate outcome measures. The latter included: TTR, percentage of INR-measurements within the target range and the variance (standard deviation (SD)^2^) of the INR-values. The clinical outcomes measures were major bleeding, intracranial bleeding, gastrointestinal bleeding, stroke, systemic embolism and death.

The definition of major bleeding was: acute posthaemorrhagic anaemia, haemothorax, recurrent and persistent haematuria, menopausal and other perimenopausal bleedings, haemorrhage from respiratory passages, unspecified haematuria and haemorrhage not classified elsewhere. Intracranial bleeding included: subarachnoid, intracerebral and epidural haemorrhage, other non-traumatic intracranial haemorrhage, focal brain injury and traumatic subdural or subarachnoid haemorrhage. Gastrointestinal bleeding was defined by: gastric, duodenal, peptic and gastrojejunal ulcer, gastritis and duodenitis. Stroke included cerebral infarction and stroke, which was not specified as haemorrhage or infarction. Systemic embolism included arteriel embolism and thrombosis. Death was defined as all-cause death and was, therefore, not limited by cardiovascular deaths.

The observation time began on the date of the first registered INR value, and terminated on the date of the first occurred clinical outcome for each outcome category, or the last registered INR value, whichever came first. Deaths recorded within 6 weeks after the last INR measurement were included in the analysis. Discontinuation from PSM was considered if the time lapse between two INR values was exceeding 6 weeks, hence person-time was censored in that case.

### Data collection

Data was obtained from nationwide registers, medical records and local databases at Aarhus University Hospital and Aalborg University Hospital.

Trained nurses or patients using an online system developed for OAT have typed INR treatment data into the two databases prospectively, and a retrieval of aggregated data was collated into a spreadsheet (Microsoft Excel, Microsoft Corp., Redmond, WA, USA). Data originating from medical records was entered in EPIDATA (Epidata software version 3.1, Epidata Association, Denmark) before conversion into the spreadsheet. The collected information included: civil registration number, sex, clinical indication for anticoagulant therapy, VKA history, date of the exam.

Finally, the compiled data was linked to three nationwide registers. The Danish National Patient Register was used for obtaining data on co-morbidity, thromboembolism and bleeding events at baseline and in the follow-up period. This register contains information on all hospital admissions in Denmark since 1977, hospital dates of admission and discharge, surgical procedures, and up to 20 discharge diagnoses coded by physicians according to the International Classification of Diseases (ICD) [18]. Data on death was obtained from the Danish Civil Registration System, which contains records of date of birth, emigration, and death for all Danish residents [19]. Data on comorbid medications was obtained from the Danish National Prescription Registry using the Anatomical Therapeutic Chemical (ATC) Classification. This registry contains information on all prescription drugs sold in Denmark since 1994 [20].

### Statistics

Characteristics of patients at baseline are presented as proportions for discrete variables and means (SD) for continuous variables. Co-morbidities were calculated according to Charlson's co-morbidity index and classified into three groups; low, medium or high co-morbidity, depending on their index value [21]. Index value 0 was classified as low, whereas 1–2 or 2<were classified as medium or high, respectively.

All aberrant therapeutic INR ranges were standardized to the predominant individual target ranges, 2 – 3 and 2.5 – 3.5. Thus, INR target ranges within 1.5 – 2.4 and 2.5 – 4.2 was standardized to 2 – 3 and 2.5 – 3.5 respectively. TTR was calculated according to Rosendaal's method [22]. The percentage of INR measurements within range was calculated for each patient, and the mean percentage is presented. The variance of INR values was calculated as a mean of all patients intra-patient variation.

Clinical endpoints are described using incidence rates and hazard rate ratios between females and males. A Cox proportional-hazards model was used for estimating the effect of sex for each outcome. A supplementary adjusted analysis was performed to evaluate the effect of sex conditional on comparable baseline characteristics. Exact 95% confidence intervals (CI) were used and a two-sided P-value <0.05 was considered statistically significant. All analyses were performed using SAS software, version 9.3 (SAS Institute), and STATA software, version 12 (StataCorp LP, TX, USA).

## Results

### Study population

During the period from the 1^st^ of June 1996 to the 31^st^ of December 2012, a total of 2,186 self-managed patients were identified at Aarhus University Hospital (1,405 patients) and Aalborg University Hospital (781 patients). A total of 118 patients were excluded for two reasons: firstly, 100 patients discontinued PSM with less than 2 registered INR measurements or a time lapse exceeding 6 weeks between the first and the second INR measurement. Secondly, 18 patients currently affiliated to Aalborg University Hospital, were identified with a previous terminated PSM treatment course at Aarhus University Hospital. After the exclusion, the study cohort comprised 2,068 patients spanning 6,900 patient-years, including 1,383 patients affiliated to Aarhus University Hospital and 685 patients affiliated to Aalborg University Hospital.

**Figure 1:**
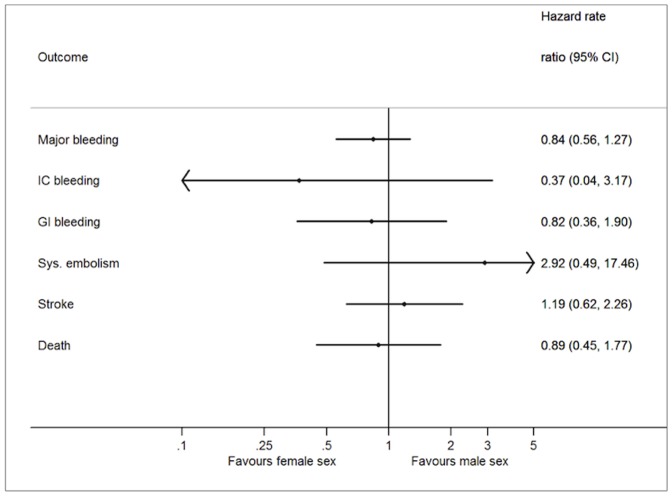
Patient self-management study flow. Abbreviations: INR: International Normalized ratio. PSM: Patient Self-management.

A flow chart of the study patients is displayed in Figure 1.

Patient demographics and baseline characteristics are summarized in Table 2. The ratio of female to males was one to two (698 women vs. 1,370 men), and significant differences were seen in age, indication and duration of prior VKA therapy. However, co-morbidities at baseline and previous complications did not differ.

**Table 2:** Baseline characteristics of patients performing self-management of oral anticoagulant therapy (N = 2068).

Variable	Female	Male	p-value
In percent, (N)	33.8 (698)	66.3 (1370)	<0.0001
Age, years mean (SD)	49.4 (14.85)	55.2 (14.06)	<0.0001
Indication for VKA therapy, % (N)			
Atrial fibrillation	19.1 (133)	31.5 (431)	0.0078
Mechanical heart valve	27.2 (190)	43.6 (597)	0.0001
Venous thromboembolism	48.9 (341)	21.7 (297)	<0.0001
Other	4.9 (34)	3.3 (45)	0.8230
Duration of prior VKA therapy*, months mean (SD)	150.1 (151.2)	130 (152.5)	0.0047
VKA medication, % (N)			
Warfarin	93.8 (655)	91.0 (1247)	0.0390
Phenprocoumon	6.2 (43)	9.0 (123)	0.7975
Previous bleeding, % (N)			
Major bleeding	10.6 (74)	9.8 (134)	0.9581
Intracranial bleeding	0.3 (2)	0.9 (12)	<0.0001
Gastrointestinal bleeding	4.3 (30)	4.9 (66)	0.6869
Previous thromboembolism, % (N)			
Stroke	10.6 (74)	9.8 (134)	0.9581
Systemic embolism	3.6 (25)	0.8 (11)	0.5231
Concomitant pharmacotherapy, % (N)			
Amiodarone	1.0 (7)	3.1 (43)	0.3625
ARB or ACE inhibitor	18.6 (130)	37.1 (508)	0.0001
Aspirin	11.5 (80)	18.0 (247)	0.2282
Beta-blocker	13.9 (97)	23.9 (328)	0.0490
H2-receptor antagonist	0.6 (4)	0 (0)	-
NSAID	4.9 (34)	3.3 (45)	0.8230
Proton-pump inhibitor	7.2 (50)	5.9 (81)	0.9334
Statin	16.1 (112)	28,3 (388)	0.0125
Co-morbidity at baseline†, % (N)			
Low	50.3 (351)	48.1 (659)	0.5506
Medium	37.7 (263)	39.3 (539)	0.7071
High	12.0 (84)	12.5 (172)	0.9335

*)Missing information on 163 patients, excluded in calculation.

^†^)Calculated according to the Charlson Comorbidity Index, group: low: 0, med: 1–2, high:>2.

Abbreviations:

N: Number.

SD: Standard deviation.

VKA: Vitamin K antagonist.

ARB: Angiotensin II receptor blocker.

ACE: Angiotensin-converting-enzyme.

NSAID: Non-steroidal anti-inflammatory drug.

### Therapeutic INR control

A full report of INR values could be obtained for all patients affiliated to Aarhus University Hospital. No INR data was found at Aalborg University Hospital prior to year 2010, due to the implementation of a new database that year. Therefore, the obtained INR measurements, and consequently the observation time for patients affiliated to Aalborg, began at the onset of PSM or earliest 1 January, 2011. The total number of INR measurements was 354,045.

The managed therapeutic INR control is provided in Table 3. TTR was 71.1% and 76.4% for females and males, respectively (p<0.0001). The percentage of INR measurements within the target range was 67.7% for females and 72.6% for males (p<0.0001). The across-patient mean intra-patient variance of INR measurements was 0.39 for females and 0.30 for males (p<0.0001).

**Table 3:** Therapeutic control of the oral anticoagulant therapy in self-managed patients (N = 2068).

Variable	Female	Male	p-value
Therapeutic INR range†, % (N)			
2.0 – 3.0*	86.4 (603)	87.8 (1203)	0.4350
2.5 – 3.5**	13.6 (95)	12.2 (167)	0.8896
Number of INR measurements per patient/year, mean (SD)	51.0 (14.2)	50.5 (17.0)	0.5312
Variance (SD^2^), mean (95% CI)	0.39 (0.36–0.41)	0.30 (0.29–0.32)	<0.0001
Mean time within therapeutic INR target range %, mean (95% CI)	71.1 (69.9–72.3)	76.4 (75.6–77.2)	<0.0001
Mean time within therapeutic INR target range %			
Crude difference	5.27 (3.88–6.65)		<0.0001
Adjusted difference***	3.56 (2.14–4.97)		<0.0001
Overall number of INR measurements within target range, %	67.7	72.6	<0.0001
Overall number of INR measurements above target range, %	16.9	14.8	<0.0001
Overall number of INR measurements below target range, %	15.5	12.6	<0.0001

^†^98 patients changed target twice, 13 patients changed target three times, 2 changed target four times.

*70 targets within 1.5 – 2.4 was standardised to 2 – 3.

**58 targets within 2.5 and 4.2 was standardised to 2.5 – 3.5.

***Adjusted for evaluating the effect of sex.

The percentage of time within therapeutic INR target range was calculated according to Rosendaal's method for each patient, and the crude and adjusted mean percentage is presented.

Abbreviations

N: Number.

INR: International Normalized Ratio.

SD: Standard deviation.

CI: Confidence Interval.

### Clinical outcome measures

**Figure 2:**
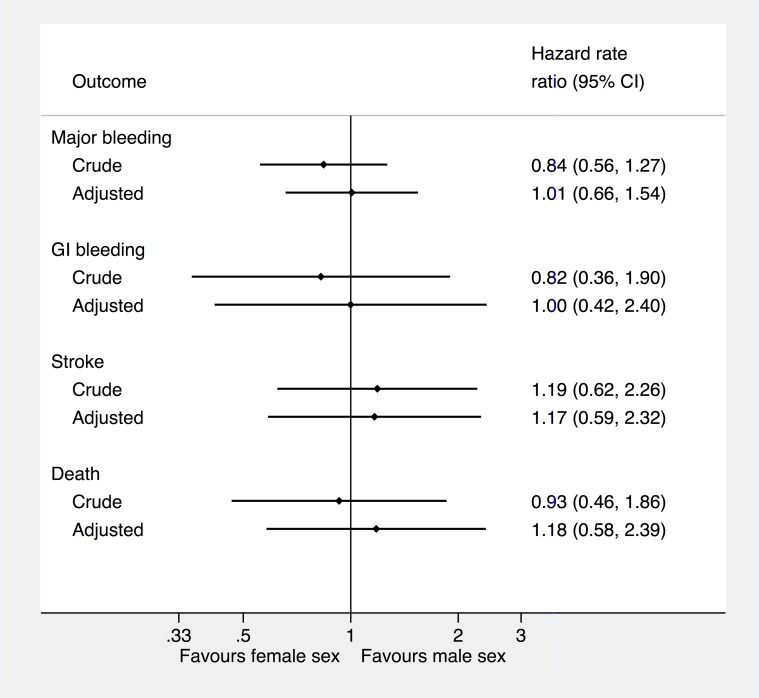
Hazard rate ratio for male sex based on Cox proportional-hazards model for each outcome. Adjusted hazard ratio optained by adjusting for age, primary indication, type of Vitamin K antagonist treatment, baseline use of ARB or ACE inhibitors, beta-blocker, and statin. The Intracranial bleeding (n = 6) and the systemic embolism (n = 5) has not been included in the analysis due to the low number of cases. Abbreviations: CI: Confidence interval. IC: Intracranial. GI: Gastrointestinal. Sys: Systemic.

The number of events and incidence rate of complications are shown in Table 4. The hazard rate ratio between females and males are shown in Figure 2. Females experienced more complications in terms of thromboembolic events, whereas males experienced more intracranial bleeding events. However, no significant differences between females and males were seen for the clinical outcome measures.

**Table 4:** Adverse events in patients on self-managed oral anticoagulant therapy (N = 2068).

Primary outcome	Female	Male	p-value
Major bleeding			
Events, % (N)	4.7 (33)	5.4 (74)	
Total person year at risk	2272.9	4293.7	
Incidence pr. 100 pt-ys (95% CI)	1.45 (1.00–2.04)	1.72 (1.35–2.16)	0.4121
Intracranial bleeding*			
Events, % (N)	0.1 (1)	0.37 (5)	
Total person year at risk	2388.7	4488.4	
Incidence pr. 100 pt-ys (95% CI)	0.04 (0.00–0.23)	0.11 (0.04–0.26)	0.3526
Gastrointestinal bleeding			
Events, % (N)	1.2 (8)	1.3 (8)	
Total person year at risk	2370.0	4455.0	
Incidence pr. 100 pt-ys (95% CI)	0.34 (0.15–0.67)	0.40 (0.24–0.64)	0.6718
Systemic embolism			
Events, % (N)	0.4 (3)	0.15 (2)	
Total person year at risk	2390.3	4498.0	
Incidence pr. 100 pt-ys (95% CI)	0.13 (0.03–0.37)	0.05 (0.01–0.16)	0.2347
Stroke			
Events, % (N)	2.2 (15)	1.8 (24)	
Total person year at risk	2358.0	4437.8	
Incidence pr. 100 pt-ys (95% CI)	0.64 (0.36–1.05)	0.54 (0.35–0.80)	0.6215
Death from any cause			
Events, % (N)	1.7 (12)	1.8 (24)	
Total person year at risk	2393.5	4507.8	
Incidence pr. 100 pt-ys (95% CI)	0.50 (0.26–0.88)	0.53 (0.36–0.79)	0.8807

*) Includes traumatic and non-traumatic intracranial bleeding.

Abbreviations:

N: Number.

Pt-ys: Patient years.

CI: Confidence interval.

## Discussion

This is the first study comparing the sex-dependent effectiveness of OAT managed as PSM in everyday clinical practice. The main results is that males have a significant higher TTR than females, whereas the incidence of death and major complications was low and essentially similar in both sexes. We found no significant difference in outcome with crude analysis compared to the adjusted analysis.

The finding of males having a higher TTR than females might be attributable to the significant differences in the baseline distribution of patient characteristics. There was a significant sex difference in the clinical indications for therapy, a vast majority of the mechanical heart valve patients were males, whereas most patients with venous thromboembolism were females. As the efficacy of PSM varies with the clinical indication for therapy and mechanical heart valves patients appear to benefit most, this can be a contributory cause to the results favouring males [8], [10]. On the contrary, females were significantly younger at baseline, thus representing an advantage, since there is an association between age and clinical outcome [10].

TTR is a predictor of the clinical outcome, but it is merely a surrogate parameter, and may not reflect the risk of clinical complications sufficiently [23], [24]. This may explain the non-significant findings in clinical outcome.

Little information regarding the sex-dependent outcome is available. Importantly, the present results are in accordance with a meta-analysis which found a small but significant sex-difference in clinical outcome, favoring the males [10]. Because the meta-analysis did not report any INR data, comparing the level of therapeutic control between the sexes is not possible.

The overrepresentation of male subjects in the current study is noteworthy and consistent with previous studies on PSM, reporting at most a fourfold more males than females [10], [25], [26]. The reasons for this imbalance are unknown, and in comparison, the ratio of males to females receiving VKA in the Danish population is more evenly divided about 60% to 40% (www.medstat.dk). General practitioners and hospital departments have referred all enrolled patients and it cannot be explained whether females are reluctant to the concept of PSM, or if females do not receive the opportunity of PSM to the same extent as males.

Since the definitions of major complications are inconsistent, a comparison of incidence rates between trials is difficult, but the low incidence rates of this study are line with previous studies, reporting incidence rates on major thromboembolism and major bleedings at 0–1.6 and 0.6–1.3 per 100 patient-years, respectively [12], [14], [27]. Further, we did not have data on complications not requiring hospital care, which may contribute to underreporting of events in this study. The all-cause mortality was low with 0.50 deaths and 0.53 per 100 patient-years in females and males, respectively. The young population with little comorbidity may have affected the results in a positive direction.

The level of therapeutic control observed for both sexes in the current study match well with that of RCT with self-managed patients achieving a TTR of 64 – 79%, and keeping 59 – 68% of their INR determinations within the range [25]–[28]. This suggests that the level of therapeutic control demonstrated in RCT can be maintained outside trial conditions. Moreover, the present results are concordant with small studies on the effectiveness of PSM [13]–[16]. Importantly, the anticoagulant control in both conventional care and PSM varies extensively among the study populations, and a comparison should therefor be interpreted with caution. Nevertheless, the low incidence rates of adverse events and the high level of INR control in the present study indicate that the efficacy of PSM achieved in RCT can be translated into everyday clinical practice showing effectiveness and efficacy is coincident. This has also been found by Nagler et al [12].

Our study has some limitations that should be acknowledged. The low age and relatively low comorbidity in our study population may reflect a pre-selection of patients. Furthermore, data is lacking on the number of patients who regretted their choice to perform PSM before passing the exam. Another major limitation is caused by the implementation of a new database in Aalborg University Hospital the 1^st^ of January 2011, which restricts the follow-up time. The implication of this is that the follow-up time do not cover the entire treatment course starting from the onset of PSM in Aalborg. However, it would not be expected that this lack of data affects the sex-dependent results. At most, it may affect the incidence of complications if the risk of complications is higher at the onset of PSM.

A multivariable adjustement model was included. However, we emphasise that this model estimates gender differences assuming males and females are alike on the included controlling factors. The main focus of the current study was however the possible overall gender difference on performance in PSM.

The primary strength of this study is the design, where local datasbases provided the treatment data and nationwide registries provided independent information on the clinical outcome. The Danish civil registration number assigned to all Danish citizens and residents enabled an unambiguous linkage on an individual level. The two center study, the unselected cohort of patients performing PSM and the long-term follow-up with 6,900 patient-years of experience are also of important strengths. A high external validity of our data is expected. All hospital care and use of equipment, including the coagulometers and strips are free of charge for the Danish patients, which might reduce the impact of socioeconomic factors.

The future of VKA is currently under discussion since the new oral anticoagulants (e.g. dabigatran, apixaban, rivaroxaban and edexaban) offer a potential advantage of requiring no monitoring of INR values. Regarding the efficacy and safety, the new oral anticoagulants compare well with VKA when managed as conventional care [29]–[31]. Whether these new drugs can match VKA treatment managed as PSM remains to be clarified. In this study, we have demonstrated that the quality of care in self-management is high in a daily clinical setting, and the new types of treatments will have to match these results in order to replace VKA in the patients able to perform PSM.

In conclusion, patient self-management of oral anticoagulant therapy outside trial conditions is clinically effective for both females and males, and results in a high TTR and a low incidence of death and major complications. Males can achieve a higher effectiveness than females in terms of TTR, but the incidence of clinical complications is similar in both sexes.
